# Clinical Phenotype of Diabetic Peripheral Neuropathy and Relation to Symptom Patterns: Cluster and Factor Analysis in Patients with Type 2 Diabetes in Korea

**DOI:** 10.1155/2017/5751687

**Published:** 2017-12-13

**Authors:** Jong Chul Won, Yong-Jin Im, Ji-Hyun Lee, Chong Hwa Kim, Hyuk Sang Kwon, Bong-Yun Cha, Tae Sun Park

**Affiliations:** ^1^Department of Internal Medicine, Cardiovascular and Metabolic Disease Center, Inje University, Sanggye Paik Hospital, Inje University School of Medicine, Seoul, Republic of Korea; ^2^Clinical Trial Center and Biomedical Research Institute, Chonbuk National University Hospital, Jeonju, Republic of Korea; ^3^Department of Internal Medicine, Daegu Catholic University School of Medicine, Daegu, Republic of Korea; ^4^Department of Internal Medicine, Sejong General Hospital, Bucheon, Republic of Korea; ^5^Department of Internal Medicine, The Catholic University of Korea, School of Medicine, Seoul, Republic of Korea; ^6^Department of Internal Medicine Chonbuk National University Medical School, Research Institute of Clinical Medicine Chonbuk National University Hospital, Division of Endocrinology and Metabolism, Jeonju, Republic of Korea

## Abstract

**Objectives:**

Patients with diabetic peripheral neuropathy (DPN) is the most common complication. However, patients are usually suffering from not only diverse sensory deficit but also neuropathy-related discomforts. The aim of this study is to identify distinct groups of patients with DPN with respect to its clinical impacts on symptom patterns and comorbidities.

**Methods:**

A hierarchical cluster analysis and factor analysis were performed to identify relevant subgroups of patients with DPN (*n* = 1338) and symptom patterns.

**Results:**

Patients with DPN were divided into three clusters: asymptomatic (cluster 1, *n* = 448, 33.5%), moderate symptoms with disturbed sleep (cluster 2, *n* = 562, 42.0%), and severe symptoms with decreased quality of life (cluster 3, *n* = 328, 24.5%). Patients in cluster 3, compared with clusters 1 and 2, were characterized by higher levels of HbA1c and more severe pain and physical impairments. Patients in cluster 2 had moderate pain levels but disturbed sleep patterns comparable to those in cluster 3. The frequency of symptoms on each item of MNSI by “painful” symptom pattern showed a similar distribution pattern with increasing intensities along the three clusters.

**Conclusions:**

Cluster and factor analysis endorsed the use of comprehensive and symptomatic subgrouping to individualize the evaluation of patients with DPN.

## 1. Introduction

Diabetic peripheral neuropathy (DPN) is the most common and earliest complication in patients with type 2 diabetes. Our previous study found that nearly one-third patients with type 2 diabetes have DPN in Korea [1]. DPN is known as progressive damage of various types of nerve fibers, and these resulted in a broad spectrum of symptoms and signs along the course of diabetes. Recently, it is suggested that DPN could be diagnosed by its typical symptom(s) and/or sign(s) in clinical terms [2]. However, DPN is a very heterogeneous disease with differences in perception and recognition sensory symptoms among patients.

Somatic pain caused by diabetes often resulted in complications such as sleep disturbance and decreased QOL. Our previous study reported that the prevalence of painful DPN in patients with DPN in Korea is 43.1%, and pain is significantly associated with daily life, quality of sleep, and life in patients with diabetic neuropathy [3]. These have made recent treatments and clinical trials for DPN failed to show efficacy in terms of patient outcomes or quality of life (QOL) in patients with DPN [2, 4].

The aim of this study was to cluster subgroups of DPN patients according to a composite of sensory symptoms and the clinical impacts on pain severity, sleep disturbance, QOL, and their relations to intensities of patterns of symptoms identified by factor analysis.

## 2. Methods

### 2.1. Subjects and Methods

Data from a previous cross-sectional observational study conducted on patients with DPN (*n* = 1338) in 2010 were used. The study design, methods used to collect the clinical and laboratory data, and the definitions for diabetes and comorbidities (hypertension, dyslipidemia, obesity, retinopathy, and nephropathy) were described previously [1, 3]. We made the diagnosis of DPN based on (1) the medical records if the date of DPN and reasons of diagnosis were clearly described (typical symptom, signs, or both) by attending physician (already diagnosed, *n* = 1073) or (2) abnormal results in both Michigan Neuropathy Screening Instrument (MNSI) scoring (≥3) and 10 g monofilament (MF) test (2 out of 10 sites) in all studied patients by trained investigator (newly diagnosed, *n* = 265). MNSI and 10 g MF test seems to be a clinically relevant and convenient method in clinical practice and recommended as simple diagnostic methods [1, 2]. At each visit, patients completed self-reported questionnaires, including the modified Korean version of Brief Pain Inventory-Short Form (BPI-SF), with pain severity and interference scales related to their pain or discomfort in the legs or hands; the medical outcomes study (MOS) sleep scale to measure overall sleep quality using a sleep problem index (SPI); the Korean version of the EuroQOL (EQ-5D) with a standardized five-item measure of health profiles; and a visual analog scale (VAS) to assess the current health state. Medications for treatment of DPN at time of study were classified by antidepressants (duloxetine, tricyclic antidepressants), anticonvulsants (pregabalin, gabapentin), *α*-lipoic acid, *γ*-linoleic acid, and others (opioid, local preparation, and vasodilators).

### 2.2. Statistical Analysis

The hierarchical cluster analysis incorporated scores from the following scales: MNSI (total score, 13) [3, 5]; BPI-SF (a 0–10 numerical scale used to measure four items of pain severity, where 0 = “no pain” and 10 = “pain as bad as you can imagine” for each severity level ([worst, least, average, and current pain]; total score, 40) [3, 6]; MOS sleep scale (a six-point scale ranging from 1 for “all of the time” to 6 for “none of the time;” the dimensions of sleep quantity [“get the amount of sleep you needed”] and sleep adequacy [“get enough sleep to feel rested upon waking in the morning”] were calculated backwards, for a total score of 36) [7, 8]; and the EQ-5D (five items with three levels indicating “no problem” (1), “some problems” (2), and “severe problems” (3), for a total score of 15) [9]. The pseudo *t*^2^ statistic was used to derive the optimal number of clusters. The number of clusters was determined through a step-down method to the final 3 clusters.

The exploratory factor analysis was performed to identify neuropathic symptom patterns using 12 of 15 items of MNSI self-administered questionnaire (excluding 3 questions: questions number 4 and number 10 were considered to be impaired circulation and general asthenia, respectively; question number 9 was not a questionnaire for neuropathic symptom but diagnosis of DPN). Principal component analysis was used to extract summary factors, and we extracted the factors with eigenvalues ≥ 1. The varimax rotation method was performed to simplify the interpretations of summary factors; one symptom was considered to be loaded on a factor if its factor loading was ≥0.40. Finally, the label of a summary factor was denominated according to the loaded symptoms on a specific factor. Descriptive statistical analyses were performed using SPSS Statistics for Windows, version 18.0 (SPSS Inc., Chicago, IL, USA) and SAS 9.4 (SAS Institute, Cary, NC, USA); significance was set at *P* < 0.05. Continuous variables are presented as means ± SDs and categorical data as frequencies and percentages and compared between clusters by ANOVA tests or chi-square or Fisher's exact tests.

## 3. Results

### 3.1. Cluster Analysis Identified 3 Groups in Patients with DPN

The mean patient age was 62.3 ± 10.7 years; 55.7% of the patients were women, with a mean body mass index of 25.0 ± 3.6 kg/m^2^. Most patients (56.9%) had taken oral hypoglycemic agent(s) for their diabetes. With cluster analysis to determine constellations of the entire patients with DPN, three clusters were identified (Table 1). Patients in cluster 3 (*n* = 328 [24.5%], “severe symptoms and decreased QOL” were characterized by higher HbA1c levels, more prevalent retinopathy and nephropathy, a higher pain level, and more severe mental and physical impairments compared with patients in cluster 2 (*n* = 562 [42.0%], “moderate symptoms and disturbed sleep”) and cluster 1 (*n* = 448 [33.5%], “asymptomatic”), as indicated by the mean comorbidity scores for all examined areas. Patients in cluster 2 had moderate pain levels but disturbed sleep patterns comparable to those of the patients in cluster 3. Patients in cluster 1 had mild pain and nearly normal sleep patterns and QOL compared with patients in clusters 2 and 3. While the pain interference index increased from clusters 1 to 3, SPI was significantly compromised in clusters 2 and 3 compared with cluster 1. The EQ-5D index was the lowest in cluster 3, and the EQ-5D VAS score decreased significantly from clusters 1 to 3.

When we performed cluster analysis in subgroups according to either already or newly diagnosed to DPN at time of study, three clustered groups were found in each subgroup. And the demographic and clinical characteristics in each subgroup analyses were showed similar trends as those in the entire population (Tables 2 and 3), while mean value of HbA1c and prevalence of retinopathy and nephropathy were statistically different along the cluster 1 to 3 in already-diagnosed patients but not in newly diagnosed patients (Tables 2 and 3).

At time of study, 65.1% (*n* = 872), 77.5% (*n* = 831), and 15.7% (*n* = 41), respectively, in the entire, already, and newly diagnosed patients were prescribed medication(s) for their DPN. While patients taking antidepressants and anticonvulsants were more prevalent in cluster 2 and 3, respectively, compared to cluster 1, those taking *α*-lipoic acid were more prevalent in cluster 1 compared to cluster 2 and 3 (Tables 1 and 2).

### 3.2. Factor Analysis Revealed Three Distinct Symptom Patterns in Patients with DPN

Three Eigenvalues were greater than unity (≥1.017) and this determined three factors computed in the entire patients.  Table 4 presented the results of factor analysis which explained 43.4% of the total variance in the 1338 patients with DPN. Factor 1 (“painful”) included number 1 (“are your legs and/or feet numb?”), number 2 (“do you ever have any burning pain in your legs and/or feet?”), number 3 (“are your feet too sensitive to touch?”), number 5 (“do you ever have any prickling feelings in your legs or feet?”), and number 6 (“does it hurt when the bed covers touch your skin?”), and number 11(“are your symptoms worse at night?”). Factor 2 (“insensate”) was number 7 (“when you get into the tub or shower, are you able to tell the hot water from the cold water?”) and number 13 (“are you able to sense your feet when you walk?”), and factor 3 (“ulcerative”) was number 8 (“have you ever had an open sore on your foot?”), number 12 (“do your legs hurt when you walk?”), and number 15 (“have you ever had an amputation?”). The total variance for each factor was 19.8%, 12.1%, and 11.5% from factor 1, 2, and 3, respectively. The factorability was proved by a Kaiser-Meyer-Olkin index that was 0.749 and the result of Bartlett's test of sphericity indicated no identity (*P* < 0.001).

### 3.3. Frequency of Symptom Patterns according to 3 Cluster Groups of DPN

**Figure 1:**
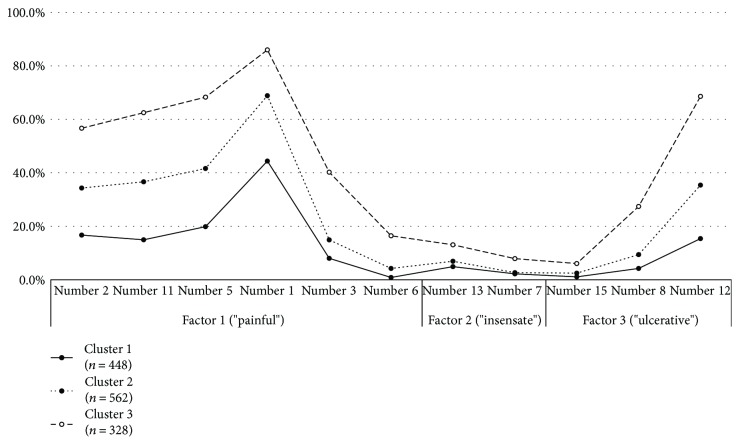
The frequency (%) distribution of symptoms on the MNSI questionnaire among the three cluster according to three subgroups of symptom patterns. MNSI number 14 (“is the skin on your feet so dry that it cracks open?”) was deleted in this figure because factor loading was <0.40 in Table 2.

Figure 1 shows the frequencies of symptoms represented by results of factor analysis on the MNSI items in the three clustered groups. The symptoms showed a similar distribution pattern but different intensities. “Painful” symptoms were increased in prominence from clusters 1 to 3 (all MNSI items, *P* < 0.05, between groups), but “insensate” was the prominent symptom that differentiated cluster 3 (highly prominent) from clusters 1 and 2 (*P* < 0.05). “Insensate” did not differ among the clusters 1 and 2. “Ulcerative” was significantly different between cluster 1and 2 and cluster 3. However, MNSI number 15 asking history of foot ulcer (“have you ever had an amputation?”) was not different between cluster 1 and 2, but those in cluster 3 were significantly more frequent in cluster 3 compared to cluster 1 and 2.

## 4. Discussion

In the current study, patients with DPN were divided into three groups by combining subjective DPN symptoms with the clinical impacts of DPN on pain, sleep, and QOL using cluster analysis in the entire patients as well as in separated analyses by diagnostic methods for DPN. Patients in cluster 3 were associated with the greatest pain intensity and the lowest QOL, whereas patients in cluster 2 were associated with moderate pain intensity but a high degree of sleep impairment. The higher level of HbA1c and proportion of patients taking insulin with or without oral hypoglycemic agent(s) in cluster 3 showed simultaneous relationships between poorly controlled glycemia and other microvascular complications: retinopathy and nephropathy. While the frequency distributions of each symptom on the MNSI were similar, more patients in cluster 3 than in clusters 1 and 2 exhibited high pain intensities and these tendencies were consistent through patterns of symptom. These results are consistent with previous observations in a large cohort of patients with the same etiology, in that patients exhibiting heterogeneous symptoms could be divided into several subgroups according to neuropathy-related symptom profiles [10, 11].

In this study, cluster 3 had prominent intensities across the subgroup of symptom patterns and, in particular, “insensate” and some items of “ulcerative” were differentiated from cluster 1 and 2. These findings are consistent with that symptoms of DPN are heterogeneous and vary widely, depending on patterns of nerve damage by different size and function. And it is suggested that pain is progressively reduced after a long-lasting painful episode to be insensible to cold, warm, and painful stimuli [12]. However, subjective symptoms are not clearly divided but even confused in patients with DPN across the duration of DPN, and it does not completely take into account different pathogenesis involving peripheral nerve damage.

In this study, pain intensity seemed to be the most important variable in the differentiation of patients with DPN into the three clusters. In addition, while the level of impaired QOL was important for differentiating cluster 2 from cluster 3, the level of sleep impairment was for differentiating cluster 1 from cluster 2. It is common for patients with DPN to experience various symptoms at the same time for which one treatment may reduce the severity of some of the symptoms but not all of the symptoms. Recently, validated patient-reported outcome measures were developed to estimate the response of patients with DPN to treatment [10, 13]. However, clinical trials investigating the efficacy of pharmacologic treatments failed to meet their primary outcomes [14], because neuropathy-related symptoms were complex and related to pain as well as psychological and physical performance, sleep quality and quantity, and overall QOL in daily activities [3, 7, 15]. Therefore, a comprehensive approach regarding the impact of DPN on these health-related issues is needed and it is important for physician when dealing with neuropathy-related symptoms in patients with type 2 diabetes to consider not only somatic symptoms but also comorbidities.

Although various measures of the clinical impacts of DPN on daily pain, sleep, and QOL were evaluated in a large number of patients, this study has several limitations. First, the diagnosis of DPN was based on definition used in clinical practice (*“possible”* to *“probable”*) rather than neurophysiological studies (*“confirmed”*) [2]. However, we thought this detection methods could be acceptable for this population to actual data for prevalence and clinical characteristics of DPN in Korea with this study. Second, the symptoms were based only on the dichromatic responses to MNSI items in this study, compared with other studies using numeric scales on self-rated questionnaires [12, 13]. Third, the effects of medications on neuropathic symptoms, pain intensity, sleep, and QOL and the difference in treatment at each cluster (*α*-lipoic acid in cluster 1, antidepressants in cluster 2, and anticonvulsants in cluster 3) were not conclusive because of the cross-sectional nature of this study. However, these complementary and exploratory analyses further support the idea that sensory phenotyping might lead to more stratified and individualized treatment in patients with DPN. Finally, how to define the pathophysiology-based differentiation of symptom clustering is not clear, and the causal relationships between comorbidities and the clustered subgroups cannot be explored because of limited cross-sectional observational study designs. We thought this study as a preliminary analysis to be a basis for further studies with detailed description and evaluation of symptoms and comorbidities, that is, mood disorder and socioeconomic aspects and various measures for outcomes. Future studies are needed to explore the effects of DPN on pain, sleep disturbance, and impaired QOL.

In conclusion, with cluster and factor analysis, we identified 3 cluster groups based on the sensory symptoms and comorbidities and 3 patterns of sensory symptoms in patients with DPN in Korea. Although, DPN is considered as heterogeneous and complex disease, these comprehensive approaches would endorse subgrouping to individualize the evaluation and treatment of patients with DPN.

## Figures and Tables

**Table 1:** Comparison of the demographic and clinical characteristics and the clinical impacts of diabetic peripheral neuropathy on pain, sleep, and quality of life in three clustered groups in the entire patients (*n* = 1338).

Variable	Cluster 1 (*n* = 448)	Cluster 2 (*n* = 562)	Cluster 3 (*n* = 328)	*P* value
Age, years	61.7 ± 9.9	62.6 ± 11.5	62.7 ± 10.5	0.3058
Female, *n* (%)	202 (45.1) ^∫,¶^	345 (61.4)	198 (60.4)	<0.001
Diabetes treatment, *n* (%)				<0.001
Diet and exercise	14 (3.1)^∫,¶^	4 (0.7)^¶^	6 (1.8)	
OHA	297 (66.3) ^∫,¶^	321 (57.1)^¶^	143 (43.6)	
Insulin	40 (8.9)^∫,¶^	76 (13.5)^¶^	70 (21.3)	
Insulin and OHA	97 (21.7)^∫,¶^	161 (28.7)^¶^	109 (33.2)	
BMI, kg/m^2^	24.9 ± 3.4	24.9 ± 3.5^¶^	25.5 ± 4.1	0.0394
FPG, mg/dL	137.8 ± 44.3^∫,¶^	141.9 ± 54.3^¶^	153.0 ± 71.1	0.0048
HbA1c, %	7.5 ± 1.3^∫,¶^	7.8 ± 1.6^¶^	8.2 ± 4.0	0.002
HbA1c, mmol/mol	58.6 ± 14.6^∫,¶^	61.3 ± 17.6^¶^	65.6 ± 43.5	0.002
Hypertension	276 (61.6)	383 (68.15)	220 (67.1)	0.0781
Dyslipidemia	242 (54.0)	301 (53.6)	161 (49.1)	0.334
Obesity	29 (6.5)	33 (5.9)	18 (5.5)	0.8408
Diabetic retinopathy, *n* (%)	122 (27.2)^∫,¶^	178 (31.7)^¶^	125 (38.1)	0.0057
Diabetic nephropathy	83 (18.5)^∫,¶^	133 (23.7)^¶^	85 (25.9)	0.0353
MNSI score	1.6 ± 1.2^∫,¶^	3.1 ± 1.7^¶^	5.4 ± 2.1	<0.001
Pain severity items				
Worst	0.39 ± 1.01^∫,¶^	3.20 ± 2.99^¶^	7.04 ± 2.41	<0.001
Weakest	0.03 ± 0.20^∫,¶^	0.60 ± 1.03^¶^	2.37 ± 2.10	<0.001
Average	0.14 ± 0.46^∫,¶^	1.76 ± 1.78^¶^	4.63 ± 2.08	<0.001
Pain interference items^∗^				
General activity	0.10 ± 0.47^∫,¶^	1.23 ± 2.19^¶^	4.96 ± 3.24	<0.001
Mood	0.21 ± 0.91^∫,¶^	1.82 ± 2.53^¶^	5.63 ± 3.10	<0.001
Walking	0.09 ± 0.51^∫,¶^	1.06 ± 2.16^¶^	4.82 ± 3.29	<0.001
Normal work	0.08 ± 0.56^∫,¶^	1.10 ± 2.13^¶^	4.73 ± 3.35	<0.001
Relationship	0.04 ± 0.31^∫,¶^	0.54 ± 1.45^¶^	3.07 ± 3.38	<0.001
Sleep	0.13 ± 0.60^∫,¶^	1.52 ± 2.57^¶^	4.22 ± 3.59	<0.001
Enjoyment of life	0.08 ± 0.42^∫,¶^	1.01 ± 2.09^¶^	3.99 ± 3.53	<0.001
Pain interference index	0.6 ± 1.5^∫,¶^	6.3 ± 6.0^¶^	17.3 ± 7.3	<0.001
MOS-SS^†^				
Sleep quantity	5.08 ± 1.26^∫,¶^	3.54 ± 1.76	3.67 ± 1.88	<0.001
Respiratory problem during sleep	5.96 ± 0.23^∫,¶^	5.67 ± 0.83	5.39 ± 1.23	<0.001
Sleep initiation problem	5.46 ± 1.10^∫,¶^	3.95 ± 1.94	4.00 ± 1.94	<0.001
Sleep maintenance problem	5.41 ± 1.12^∫,¶^	3.92 ± 1.81	4.05 ± 1.85	<0.001
Somnolence	5.77 ± 0.64^∫,¶^	5.09 ± 1.32	4.92 ± 1.40	<0.001
Sleep adequacy	5.29 ± 1.17^∫,¶^	3.84 ± 1.79	4.00 ± 1.83	<0.001
Sleep problem index	33.0 ± 3.1^∫,¶^	26.0 ± 6.3	26.0 ± 7.2	
EQ-5D^§^				
Mobility	1.07 ± 0.25^¶^	1.26 ± 0.45^¶^	1.80 ± 0.50	<0.001
Self-care	1.01 ± 0.13^¶^	1.09 ± 0.30^¶^	1.33 ± 0.54	<0.001
Usual activity	1.03 ± 0.17^¶^	1.21 ± 0.43^¶^	1.76 ± 0.55	<0.001
Pain discomfort	1.08 ± 0.29^¶^	1.51 ± 0.53^¶^	2.17 ± 0.51	<0.001
Anxiety/depression	1.11 ± 0.31^¶^	1.40 ± 0.51^¶^	1.79 ± 0.67	<0.001
EQ-5D index^§^	5.3 ± 0.7^¶^	6.5 ± 1.4^¶^	8.8 ± 1.8	<0.001
EQ-5D VAS^‡^	81.8 ± 9.6^∫,¶^	65.9 ± 16.1^¶^	51.4 ± 19.3	<0.001
Medications for DPN				
None	164 (36.6)	191 (34.0)	111 (33.8)	0.625
Antidepressants	30 (6.7)^∫,¶^	68 (12.1)^¶^	33 (10.1)	0.016
Anticonvulsants	47 (10.5)^∫,¶^	81 (14.4)^¶^	72 (22.0)	<0.001
*α*-Lipoic acid	107 (23.9)^∫,¶^	116 (20.6)	54 (16.5)	0.042
*γ*-Linoleic acid	34 (7.6)	33 (5.9)	27 (8.2)	0.351
Others	83 (18.5)	102 (18.2)	54 (16.5)	0.739

Data are expressed as means ± SD for continuous variables and frequency (%) for categorical variables. ^∗^Items were derived from the BPI-SF. A 0–10 numeric rating scale was anchored at 0 for “no pain” and 10 for “pain as bad as you can imagine.” ^†^Item response on a 6-point scale ranging from 1 for “all of the time” to 6 for “none of the time;” dimensions of sleep quantity, “get the amount of sleep you needed;” and sleep adequacy, “get enough sleep to feel rested upon waking in the morning” were calculated backwards. ^§^Items were from three levels indicating “no problem” (or 1), “some problems” (or 2), and “severe problems” (or 3), and EQ-5D index was the sum of scores of 5 dimensions. ^‡^Values from 0 to 100, where 0 represents the worst imaginable health state and 100 represents the best imaginable health state. ^∫^*P* < 0.05 versus ≤cluster 2 and ^¶^*P* < 0.05 versus cluster 3. OHA: oral hypoglycemic agent(s); BMI: body mass index; FPG: fasting plasma glucose; MNSI: Michigan Neuropathy Screening Instrument questionnaire; MOS-SS: medical outcomes study sleep scale; EQ-5D: EuroQol, 5-dimensions; VAS: visual analog scale.

**Table 2:** Comparison of the demographic and clinical characteristics and the clinical impacts of diabetic peripheral neuropathy on pain, sleep, and quality of life in three clustered groups in already diagnosed patients (*n* = 1073).

Variable	Cluster 1 (*n* = 542)	Cluster 2 (*n* = 407)	Cluster 3 (*n* = 124)	*P* value
Age, years	61.2 ± 10.7	62.7 ± 10.9	60.3 ± 11.2	0.030
Female, *n* (%)	267 (49.3)	245 (60.2)	79 (63.7)	<0.001
Diabetes treatment, *n* (%)				<0.001
Diet and exercise	12 (2.2)	4 (1.0)	0 (0.0)	
OHA	347 (64.0)	203 (49.9)	49 (39.5)	
Insulin	54 (10.0)	67 (16.5)	26 (21.0)	
Insulin and OHA	129 (23.8)	133 (32.7)	49 (39.5)	
BMI, kg/m^2^	24.8 ± 3.4	25.0 ± 3.7	25.5 ± 3.8	0.165
FPG, mg/dL	141.6 ± 49.8	141.7 ± 52.0	164.2 ± 89.6	0.001
HbA1c, %	7.6 ± 1.5	7.7 ± 1.5	8.3 ± 2.1	<0.001
HbA1c, mmol/mol	60.0 ± 16.6	60.5 ± 16.4	67.0 ± 22.5	<0.001
Hypertension	339 (62.6)	277 (68.1)	87 (70.2)	0.107
Dyslipidemia	294 (54.2)	217 (53.3)	61 (49.2)	0.596
Obesity	42 (7.8)	19 (4.7)	12 (9.7)	0.070
Diabetic retinopathy, *n* (%)	162 (29.9)	155 (38.1)	55 (44.4)	0.002
Diabetic nephropathy	109 (20.1)	112 (27.5)	33 (26.6)	0.021
MNSI score	1.8 ± 1.6	3.6 ± 1.9	5.8 ± 2.4	<0.001
Pain severity items				
Worst	0.6 ± 1.4	4.9 ± 2.8	7.8 ± 1.9	<0.001
Weakest	0.1 ± 0.3	1.2 ± 1.6	2.8 ± 2.1	<0.001
Average	0.3 ± 0.7	2.8 ± 1.9	5.4 ± 1.8	<0.001
Pain interference items^∗^				
General activity	0.2 ± 0.7	2.2 ± 2.8	6.2 ± 2.8	<0.001
Mood	0.3 ± 1.1	3.0 ± 3.0	7.0 ± 2.2	<0.001
Walking	0.1 ± 0.7	2.1 ± 2.9	5.7 ± 3.1	<0.001
Normal work	0.1 ± 0.7	2.0 ± 2.8	6.0 ± 3.0	<0.001
Relationship	0.1 ± 0.5	1.0 ± 2.1	4.2 ± 3.4	<0.001
Sleep	0.2 ± 0.9	2.2 ± 3.0	5.9 ± 3.3	<0.001
Enjoyment of life	0.1 ± 0.6	1.7 ± 2.6	5.4 ± 3.3	<0.001
Pain interference index	0.9 ± 2.3	10.4 ± 6.5	20.4 ± 6.2	<0.001
MOS-SS^†^				
Sleep quantity	4.8 ± 1.5	3.7 ± 1.8	2.5 ± 1.5	<0.001
Respiratory problem during sleep	5.9 ± 0.4	5.7 ± 0.8	5.0 ± 1.5	<0.001
Sleep initiation problem	5.2 ± 1.4	4.1 ± 1.9	3.1 ± 1.8	<0.001
Sleep maintenance problem	5.1 ± 1.4	4.1 ± 1.8	3.3 ± 1.8	<0.001
Somnolence	5.7 ± 0.8	5.1 ± 1.3	4.5 ± 1.5	<0.001
Sleep adequacy	5.0 ± 1.4	4.0 ± 1.8	3.0 ± 1.8	<0.001
Sleep problem index	31.7 ± 4.6	26.6 ± 6.2	21.4 ± 6.2	<0.001
EQ-5D^§^				
Mobility	1.1 ± 0.3	1.4 ± 0.5	1.9 ± 0.5	<0.001
Self-care	1.0 ± 0.1	1.1 ± 0.4	1.4 ± 0.5	<0.001
Usual activity	1.0 ± 0.2	1.3 ± 0.5	2.0 ± 0.5	<0.001
Pain discomfort	1.1 ± 0.3	1.7 ± 0.5	2.4 ± 0.5	<0.001
Anxiety/depression	1.2 ± 0.4	1.4 ± 0.6	2.1 ± 0.7	<0.001
EQ-5D index^§^	5.4 ± 0.8	7.0 ± 1.5	9.7 ± 1.5	<0.001
EQ-5D VAS^‡^	77.9 ± 12.8	64.0 ± 16.1	44.2 ± 19.0	<0.001
Medications for DPN				
None	142 (26.2)	79 (19.4)	21 (16.9)	0.013
Antidepressants	50 (9.2)	60 (14.7)	17 (13.7)	0.027
Anticonvulsants	67 (12.4)	99 (24.3)	31 (25.0)	<0.001
*α*-Lipoic acid	148 (27.3)	91 (22.4)	27 (21.8)	0.154
*γ*-Linoleic acid	47 (8.7)	29 (7.1)	14 (11.3)	0.323
Others	113 (20.9)	80 (19.7)	26 (21.0)	0.891

Data are expressed as means ± SD for continuous variables and frequency (%) for categorical variables. ^∗^Items were derived from the BPI-SF. A 0–10 numeric rating scale was anchored at 0 for “no pain” and 10 for “pain as bad as you can imagine.” ^†^Item response on a 6-point scale ranging from 1 for “all of the time” to 6 for “none of the time;” dimensions of sleep quantity, “get the amount of sleep you needed;” and sleep adequacy, “get enough sleep to feel rested upon waking in the morning” were calculated backwards. ^§^Items were from three levels indicating “no problem” (or 1), “some problems” (or 2), and “severe problems” (or 3), and EQ-5D index was the sum of scores of 5 dimensions. ^‡^Values from 0 to 100, where 0 represents the worst imaginable health state and 100 represents the best imaginable health state. OHA: oral hypoglycemic agent(s); BMI: body mass index; FPG: fasting plasma glucose; MNSI: Michigan Neuropathy Screening Instrument questionnaire; MOS-SS: medical outcomes study sleep scale; EQ-5D: EuroQol, 5-dimensions; VAS: visual analog scale.

**Table 3:** Comparison of the demographic and clinical characteristics and the clinical impacts of diabetic peripheral neuropathy on pain, sleep, and quality of life in three clustered groups in newly diagnosed patients (*n* = 265).

Variable	Cluster 1 (*n* = 542)	Cluster 2 (*n* = 407)	Cluster 3 (*n* = 124)	*P* value
Age, years	64.3 ± 10.0	66.1 ± 9.7	64.6 ± 9.5	0.447
Female, *n* (%)	89.0(58.6)	33.0(48.5)	32.0(71.1)	0.058
Diabetes treatment, *n* (%)				0.008
Diet and exercise	6 (4.0)	1 (1.5)	1 (2.2)	
OHA	105 (69.1)	34 (50.0)	23 (51.1)	
Insulin	14 (9.2)	18 (26.5)	7 (15.6)	
Insulin and OHA	27 (17.8)	15 (22.1)	14 (31.1)	
BMI, kg/m^2^	25.4 ± 3.5	24.5 ± 3.8	25.6 ± 4.2	0.276
FPG, mg/dL	132.4 ± 41.3	147.6 ± 51.6	141.0 ± 76.1	0.260
HbA1c, %	7.5 ± 1.4	7.9 ± 1.7	9.1 ± 9.8	0.121
HbA1c, mmol/mol	59.0 ± 15.1	62.8 ± 18.1	76.3 ± 106.7	0.121
Hypertension	101 (66.5)	48 (70.6)	27 (60.0)	0.506
Dyslipidemia	76 (50.0)	36 (52.9)	20 (44.4)	0.675
Obesity	4 (2.6)	0 (0.0)	3 (6.7)	0.096
Diabetic retinopathy, *n* (%)	27 (17.8)	18 (26.5)	8 (17.8)	0.302
Diabetic nephropathy	25 (16.5)	16 (23.5)	6 (13.3)	0.311
MNSI score	3.1 ± 1.2	3.9 ± 1.2	6.5 ± 1.9	<0.001
Pain severity items				
Worst	2.1 ± 2.7	5.0 ± 3.6	7.6 ± 2.5	<0.001
Weakest	0.4 ± 0.8	1.3 ± 1.6	2.9 ± 2.2	<0.001
Average	1.1 ± 1.5	3.1 ± 2.5	5.1 ± 2.3	<0.001
Pain interference items^∗^				
General activity	0.6 ± 1.5	3.2 ± 3.2	6.4 ± 2.9	<0.001
Mood	1.1 ± 2.0	3.7 ± 3.2	6.8 ± 2.9	<0.001
Walking	0.6 ± 1.5	3.0 ± 3.2	6.1 ± 3.2	<0.001
Normal work	0.7 ± 1.5	2.9 ± 3.1	6.1 ± 3.3	<0.001
Relationship	0.3 ± 1.0	1.7 ± 2.5	4.8 ± 3.7	<0.001
Sleep	0.8 ± 1.8	2.1 ± 3.0	5.8 ± 3.4	<0.001
Enjoyment of life	0.5 ± 1.3	2.3 ± 3.1	5.7 ± 3.7	<0.001
Pain interference index	4.0 ± 5.1	10.8 ± 7.9	19.4 ± 7.7	<0.001
MOS-SS^†^				
Sleep quantity	4.3 ± 1.7	4.2 ± 1.8	2.7 ± 1.7	<0.001
Respiratory problem during sleep	5.8 ± 0.7	5.8 ± 0.6	4.8 ± 1.7	<0.001
Sleep initiation problem	4.6 ± 1.8	4.5 ± 1.7	2.6 ± 1.7	<0.001
Sleep maintenance problem	4.4 ± 1.8	4.7 ± 1.6	2.7 ± 1.8	<0.001
Somnolence	5.4 ± 1.0	5.3 ± 1.1	3.8 ± 1.7	<0.001
Sleep adequacy	4.6 ± 1.6	4.5 ± 1.7	2.7 ± 1.8	<0.001
Sleep problem index	29.2 ± 5.9	28.9 ± 5.4	19.3 ± 5.8	<0.001
EQ-5D^§^				
Mobility	1.2 ± 0.4	1.8 ± 0.5	2.0 ± 0.5	<0.001
Self-care	1.1 ± 0.3	1.2 ± 0.5	1.6 ± 0.7	<0.001
Usual activity	1.1 ± 0.3	1.7 ± 0.5	2.0 ± 0.5	<0.001
Pain discomfort	1.3 ± 0.5	2.0 ± 0.4	2.4 ± 0.6	<0.001
Anxiety/depression	1.3 ± 0.5	1.6 ± 0.5	2.0 ± 0.6	<0.001
EQ-5D index^§^	6.0 ± 1.4	8.2 ± 1.2	10.0 ± 1.9	<0.001
EQ-5D VAS^‡^	74.2 ± 14.1	53.9 ± 16.7	41.4 ± 21.2	<0.001
Medications for DPN				
None	133 (87.5)	55 (80.9)	36 (80.0)	0.298
Antidepressants	1 (0.7)	3 (4.4)	0 (0.0)	0.071
Anticonvulsants	1 (0.7)	1 (1.5)	1 (2.2)	0.653
*α*-Lipoic acid	6 (4.0)	4 (5.9)	1 (2.2)	0.622
*γ*-Linoleic acid	1 (0.7)	1 (1.5)	2 (4.4)	0.187
Others	10 (6.6)	5 (7.4)	5 (11.1)	0.598

Data are expressed as means ± SD for continuous variables and frequency (%) for categorical variables. ^∗^Items were derived from the BPI-SF. A 0–10 numeric rating scale was anchored at 0 for “no pain” and 10 for “pain as bad as you can imagine.” ^†^Item response on a 6-point scale ranging from 1 for “all of the time” to 6 for “none of the time;” dimensions of sleep quantity, “get the amount of sleep you needed;” and sleep adequacy, “get enough sleep to feel rested upon waking in the morning” were calculated backwards. ^§^Items were from three levels indicating “no problem” (or 1), “some problems” (or 2), and “severe problems” (or 3), and EQ-5D index was the sum of scores of 5 dimensions. ^‡^Values from 0 to 100, where 0 represents the worst imaginable health state and 100 represent the best imaginable health state. OHA: oral hypoglycemic agent(s); BMI: body mass index; FPG: fasting plasma glucose; MNSI: Michigan Neuropathy Screening Instrument questionnaire; MOS-SS: medical outcomes study sleep scale; EQ-5D: EuroQol, 5-dimensions; VA: visual analog scale.

**Table 4:** Varimax rotated factor loadings for Michigan Neuropathy Screening Instrument (MNSI) questionnaire items.

MNSI items	Factor	Factor	Factor
	1	2	3
Number 2. Do you ever have any burning pain in your legs and/or feet?	**0.662**	0.076	−0.057
Number 11. Are your symptoms worse at night?	**0.648**	−0.003	−0.030
Number 5. Do you ever have any prickling feelings in your legs or feet?	**0.609**	0.014	0.158
Number 1. Are your legs and/or feet numb?	**0.566**	−0.068	0.125
Number 3. Are your feet too sensitive to touch?	**0.536**	0.020	0.119
Number 6. Does it hurt when the bed covers touch your skin?	**0.410**	0.351	0.037
Number 14. Is the skin on your feet so dry that it cracks open?	0.263	−0.042	0.202
Number 13. Are you able to sense your feet when you walk?	−0.058	**0.769**	0.063
Number 7. When you get into the tub or shower, are you able to tell the hot water from the cold water?	0.022	**0.758**	0.050
Number 15. Have you ever had an amputation?	−0.122	−0.021	**0.781**
Number 8. Have you ever had an open sore on your foot?	0.195	0.173	**0.654**
Number 12. Do your legs hurt when you walk?	0.334	0.010	**0.394**
Variance, %	19.8	12.1	11.5

Factor loadings ≥ 0.40 are in bold.
